# Expression of adenoviral E1A throws the PIDD switch

**DOI:** 10.1038/cddis.2016.445

**Published:** 2017-01-05

**Authors:** Jay R Radke, James L Cook

**Affiliations:** 1 Research Section, Edward Hines, Jr. VA Hospital, Hines, IL USA; 2 Division of Infectious Diseases, Loyola University Medical Center; Infectious Diseases and Immunology Research Institute, Loyola University Chicago – Stritch School of Medicine, Maywood, IL USA; 3 Department of Microbiology and Immunology, Loyola University Chicago – Stritch School of Medicine, Maywood, IL, USA

The adenoviral early region 1A gene (E1A) is a modulator of cellular and viral gene transcription and the primary mediator of cell cycle induction during infection of quiescent cells. It is through these cell cycle effects that E1A can immortalize cells during either abortive infection or stable expression. A side effect of E1A altering cell cycle and host transcriptional activity is that expression of E1A increases the sensitivity of cells to cytotoxicity induced by components of the host cellular immune response (natural killer cells, cytotoxic T cells and macrophages) and apoptosis-inducing injuries (TNF-super family members, chemotherapeutic agents and oxidative stress).^1^ As a result of cell sensitizing E1A activities, targeted expression and delivery of E1A to tumor cells has been used to treat various forms of cancer. However, the cellular pathways and mechanisms through which E1A increases cellular sensitivity to these potentially cytotoxic injuries are not well defined.

The best understood mechanisms through which E1A enhances cellular apoptotic responses involve the TNF-super family (TNF-*α*, TRAIL and Fas ligand). E1A sensitizes cells to these extrinsic apoptotic stimuli by multiple cellular effects, including induction of increased death receptor expression, increased procaspase 8 processing and inhibition of anti-apoptotic NF-*κ*B dependent transcriptional responses. E1A represses signaling-induced NF-*κ*B dependent transcription by multiple mechanisms, including inhibition of I *κ*B kinase activity, alteration of the quality of NF-*κ*B dimers in the nucleus, sequestration of the NF-*κ*B co-activator p300/CBP, and interactions with Rb family members that regulate formation of NF-*κ*B-enhancer complexes in the nucleus.^2, 3, 4^ The most convincing evidence that NF-*κ*B dependent anti-apoptotic gene expression is a critical final common pathway through which E1A expressing cells are sensitized to apoptosis is the observation that overexpression of the p65-RelA NF-*κ*B subunit effectively eliminates the apoptotic sensitivity of E1A-positive cells to TNF-super family molecules.

NF-*κ*B, as a result of its induction of anti-apoptotic gene expression, has been called the central regulator of apoptotic cell death.^5^ Evidence from studies on E1A-mediated sensitization to TNF-super family members—agents that trigger death receptor mediated ‘extrinsic' apoptotic responses—suggested the possibility that E1A repression of NF-*κ*B dependent transcription responses might also account for cellular sensitivity to agents of ‘intrinsic' apoptosis, such as DNA-damage-inducing chemotherapeutic agents, oxidative stresses and injuries by innate immune cells. Using E1A-positive cells that were either selected for resistance to TNF or that over-expressed NF-*κ*B p65/RelA, we found that NF-*κ*B dependent cellular responses are not required for E1A-induced sensitivity to macrophage-mediated nitric oxide (NO)-induced apoptosis.^6^ Furthermore, the studies revealed that NO-induced apoptosis of E1A-positive cells requires the expression and activity of caspase-2. On the basis of those results, we proposed that E1A-enhancement of NO-induced caspase-2 activation might be the direct cause of irreversible mitochondrial injury, which would then trigger apoptosis.

Although caspase-2 is a highly conserved member of the caspase family, its precise role in apoptosis has remained elusive. Caspase-2 has been linked to apoptotic cell death associated with various cellular injuries that cause metabolic imbalance, DNA damage, ER stress or mitotic stress.^7^ However, caspase-2 knockout animals do not show defects in apoptotic cell death responses to various injuries.^7^ Caspase-2 contains a caspase activation and recruitment domain (CARD), like that of other initiator caspases (such as caspase-1, -8 and -9), through which it is recruited to high molecular weight complexes, but caspase-2 has also been proposed to be an effector caspase that can be activated by caspase-3. As an initiator caspase, caspase-2 is recruited to a high molecular weight complex, call the PIDDosome, for efficient activation.^8, 9^ The PIDDosome is composed of three proteins, the p53-induced protein with a death domain (PIDD), caspase-2 and RIP-associated Ich-1/Ced-3 homologous protein with a death domain (RAIDD). Caspase-2 interacts with RAIDD through dimerization of their respective CARD domains, whereas PIDD and RAIDD associate with each other through their C-terminal death domains.^10^ In addition to these proteins, heat shock proteins 90 and 70 also interact with this complex and can regulate the activation of caspase-2 and the formation and stability of the PIDDosome. However, caspase-2 activation does not always proceed through the PIDDosome.^11^

We have recently reported evidence that supports the hypothesis that the mechanism E1A alters to increase cellular sensitivity to intrinsic apoptotic injuries involves the PIDD-Caspase-2 activation pathway. In these studies, we observed that, in addition to NO sensitization, E1A-induced sensitivity to DNA-damaging chemotherapeutic agents (etoposide and gemcitabine) is independent of NF-*κ*B activation responses but dependent on caspase-2 activity.^12^ Caspase-2 was found to be the key initiator and mitochondria-injuring caspase in this system. Furthermore, expression and cleavage of PIDD was required for caspase-2 activation, mitochondrial injury and E1A-enhanced apoptotic cell death.^12^

On the basis of these results and previous observations, we propose a new model of E1A-mediated cellular sensitization to apoptotic injury (Figure 1). Expression of E1A inhibits NF-*κ*B dependent cellular responses to proapoptotic signals, making cells highly susceptible to extrinsic apoptosis induced by members of the TNF-super family. This NF-*κ*B inhibitory effect might also block the reported PIDD-induced NF-*κ*B anti-apoptotic effect.^13^ However, this E1A repression of NF-*κ*B dependent transcription alone is insufficient to sensitize cells to intrinsic apoptosis induced by DNA-damaging chemotherapeutic agents. In addition to repressing NF-*κ*B activation responses, E1A enhances injury-induced processing of PIDD-C to PIDD-CC, thereby ‘throwing the PIDD switch' from an anti-apoptotic response to favor a caspase-2-induced apoptotic response.^13^ Definition of the E1A controlled mechanism(s) responsible for this shift in the balance of the PIDD pathway response toward caspase-2 activation, through increased PIDD processing, will require further study. We believe the answers will be found through studies of the interactions between E1A activities and the cellular mechanisms that regulate the formation and activation of the PIDDosome.

## Figures and Tables

**Figure 1:**
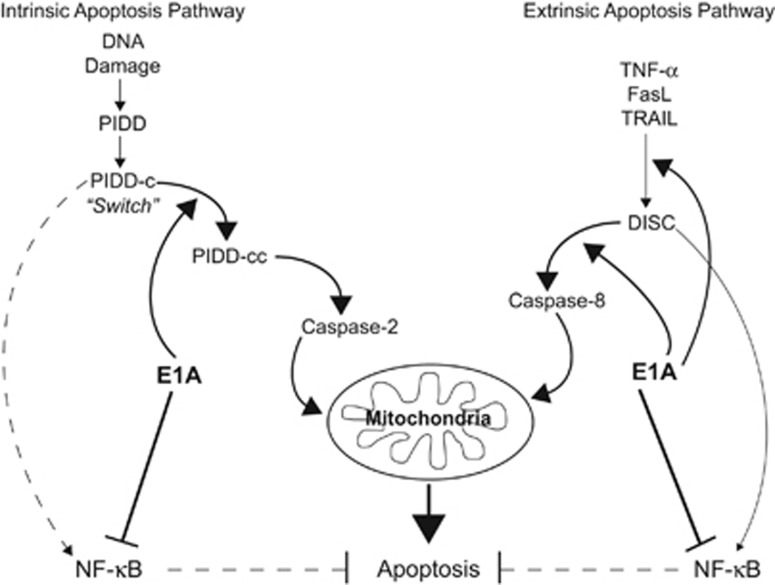
E1A ‘throws the PIDD switch' to favor caspase-2-mediated apoptosis. Expression of E1A increases cellular sensitivity to both extrinsic and intrinsic apoptosis-inducing stimuli by altering several cellular mechanisms. E1A sensitizes cells to extrinsic apoptosis induced by members of the TNF-super family (TNF-α, Fas Ligand [FasL] and TRAIL) by increasing death receptor expression and caspase 8 activation and by repressing NF-*κ*B dependent anti-apoptotic defenses. In the case of intrinsic apoptosis pathway triggering by DNA-damage-inducing stimuli, E1A might also repress the NF-*κ*B dependent response, but the essential E1A activity is enhancement of processing of PIDD-C to PIDD-CC, which pushes the balance of intrinsic pathway activities toward caspase-2-dependent mitochondrial injury and cell death
